# Effects of Amino Hyperbranched Polymer-Modified Carbon Nanotubes on the Crystallization Behavior of Poly (*L*-Lactic Acid) (PLLA)

**DOI:** 10.3390/polym14112188

**Published:** 2022-05-28

**Authors:** Bofan Shen, Shulai Lu, Chunfu Sun, Zhenbiao Song, Fuyi Zhang, Jian Kang, Ya Cao, Ming Xiang

**Affiliations:** 1 State Key Laboratory of Polymer Materials Engineering, Polymer Research Institute, Sichuan University, Chengdu 610065, China; sbf991230@163.com (B.S.); zhangfuyi_robert@foxmail.com (F.Z.); caoya@scu.edu.cn (Y.C.); xiangming@scu.edu.cn (M.X.); 2 Synthetic Resin Factory of Jilin Petrochemical Company, PetroChina, Jilin 132021, China; chunfusun@petrochina.com.cn (C.S.); zhenbiaosong@petrochina.com.cn (Z.S.); 3 PetroChina ABS Resin Technology Center, Jilin 132021, China

**Keywords:** PLLA, carbon nanotubes, crystallization behavior, hyperbranched polymer, surface modification

## Abstract

Poly-*L*-lactic acid (PLLA) is an environmentally friendly and renewable polymer material with excellent prospects, but its low crystallization rate greatly limits its application. Through the amidation reaction between amino hyperbranched polymer (HBP N103) and carboxylated carbon nanotubes (CNTs), CNTs-N103 was obtained. The modification was confirmed by Fourier-transform infrared (FTIR) spectroscopy, X-ray electron spectroscopy (XPS) and thermogravimetric analysis (TGA). Using transmission electron microscopy (TEM), we observed the changes on the surface of modified CNTs. PLLA/CNT composites were prepared, and differential scanning calorimetry (DSC) was used to investigate the crystallization behavior of the composites. The results showed that the addition of CNTs could greatly improve the crystallization properties of PLLA; at the same concentration, the modified CNTs had better regulation ability in PLLA crystallization than the unmodified CNTs. Moreover, in the concentration range of 0.1–1%, with the increase in HBP concentration, the ability of CNTs-N103 to regulate the crystallization of PLLA increased as well. Wide-angle X-ray diffraction (WAXD) once again proved the improvement of the crystallization ability. The results of polarized optical microscopy (PLOM) showed that the number of nucleation points increased and the crystal became smaller.

## 1. Introduction

Poly-*L*-lactic acid (PLLA) is a kind of polymer material obtained from renewable resources, which is widely used as an environmentally friendly packaging material [1], 3D printing material [2], biomedical material [3] and so on due to its good biodegradability, biocompatibility, non-toxicity and non-irritation as well as excellent mechanical properties [4,5]. However, the application of PLLA is greatly limited on account of its low crystallization rate and brittleness [6,7]. Therefore, the efficient regulation of PLLA’s crystallization rate has become a research hotspot. Great efforts had been made in this field and results have revealed that the crystallization rate of PLLA can be improved by copolymerization [8], blending modification [9] and the addition of a nucleating agent [10], among which the latter is the most convenient and economical method [11].

Carbon nanotubes (CNTs), because of their unique structure and performance, are often wildly used as reinforcement fillers in composite materials, and have attracted extensive attention in many fields such as nanomaterials science and biomedical materials science [12,13,14]. CNTs are known as one of the initiators of the material revolution in the 21st century, and their application prospects are very broad [14]. Previous studies have shown that CNTs can act as nucleating agents in PLLA. Wang et al. [15] used CNTs as nucleating agents of polylactic acid (PLA) to prepare CNTs/PLA composites, and the results showed that the spherulite size of PLA was significantly reduced after the addition of CNTs. However, the high surface energy of CNTs makes it impossible for them to be evenly dispersed in the composite material, and thus the composite materials do not present the expected properties [16]. Based on the above issues, the current researchers propose that the surface modification of CNTs could solve this problem.

The general modification methods include non-covalent chemical modification and covalent chemical modification [17,18]. Non-covalent chemical modification, namely physical adsorption and the coating of CNTs, will not damage the surface structure of CNTs, which can better maintain the integrity of the structure of CNTs. Covalent chemical modification uses the interactions between chemical bonds to change the surface structure of CNTs and graft functional groups, etc. [18]. To sum up, it is the focus and challenge of current research to develop new modification methods for PLLA so as to obtain better modification effects of CNTs in PLLA. 

Hyperbranched polyesters (HBP), compared with traditional linear polymers, have the advantages of a low viscosity, high solubility and a large number of terminal functional groups, and the steric hindrance effect of HBP might efficiently reduce the agglomeration of CNTs effectively [19,20]. Therefore, HBP could be potentially used as ideal modification materials for CNTs. 

Interestingly, HBP have been added to PLLA and the addition of HBP has been found to efficiently regulate the crystallization of PLLA [21,22]. Based on these studies, an interesting strategy comes out: on the one hand, HBP can be grafted onto CNTs to efficiently improve the dispersion of CNTs in the matrix; on the other hand, HBP can also be used to further regulate the crystallization of PLLA, which might achieve a more efficient regulation effect. As far as we know, this has not been reported before. The authors of this paper prepared modified CNTs by amino-terminated HBP, and then prepared PLLA/CNT composites, and comparatively studied the impacts of HBP modification on CNTs, as well as the concentration of the filler, on the crystallization behavior and morphologies of PLLA. The related mechanism is proposed.

## 2. Experimental Section

### 2.1. Materials and Sample Preparation

#### 2.1.1. Materials

**Scheme 1:**
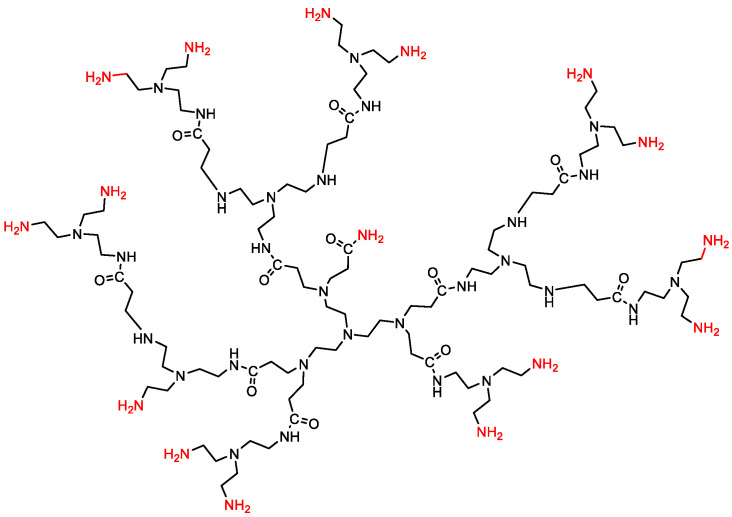
Chemical structure of HBP N103.

The average molecular weight (M_w_) of PLLA 4032D is 2 × 10^5^ g/mol, and its melting index is 5.7 g/10 min [9,23], produced by Natureworks Co., Ltd., Minnetonka, MN, USA. The hyperbranched polyester N103 was purchased from Wuhan Hyperbranched resin Technology Co., LTD, China. Its molecular weight is 1900–2200 g/mol and amino number content is 12–16 mol/mol, and its chemical structure is illustrated in Scheme 1. The carbon nanotubes used are carboxylated high-purity multi-walled carbon nanotubes, produced by Beijing Boyu High-tech New Materials Technology Co., Ltd., Beijing, China. *N*,*N*′-dicyclohexyl carbon imide (DCC) and *N*,*N*′-dimethylformamide (DMF) were purchased from Chengdu Changlian Chemical Reagent Co., Ltd., Chengdu, Sichuan, China, and ethanol was purchased from Chengdu Dingsheng Era Technology Co., Ltd., Chengdu, Sichuan, China. Deionized water was self-made in the laboratory. The phenolic antioxidant Irganox 1010 was produced by BASF China Co., Ltd., Shanghai, China.

#### 2.1.2. Preparation of Modified CNTs

**Scheme 2:**
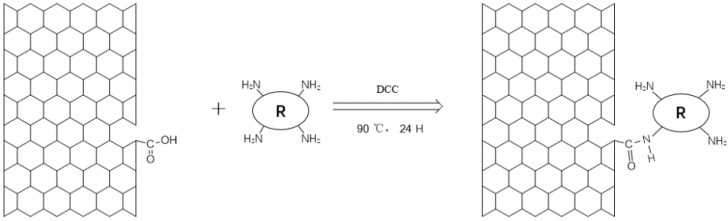
Preparation of modified CNTs and formula of HBP N103 (R(NH_2_)_4_).

HBP N103-modified CNTs were prepared by the following steps, as illustrated in Scheme 2: HBP N103 was vacuum dried at 60 °C for 24 h before use. HBP N103 (R(NH_2_)_4_ in Scheme 2) was added to the molecular sieve to absorb water and small molecular impurities, and was then vacuum dried at 60 °C for more than 24 h. We added 1 g of DCC and 1g of CNTs into a 250 mL beaker. This was stirred and dissolved in 5 g of HBP N103 in 150 mL DMF, and then CNTs were mixed with DMF solution dissolved with HBP N103. An ultrasonic dispersing machine was used to ensure it was mixed evenly. The solution was then poured into a round-bottomed flask and refluxed at 90 °C for 24 h under magnetic agitation. The product was filtered by PVDF membrane with a pore size of 0.22 μm and washed with ethanol and deionized water several times to remove the unreacted HBP N103 and the impurities generated by the reaction. Finally, CNTs modified by HBP N103 were prepared by vacuum drying at 60 °C for 24 h.

#### 2.1.3. Preparation of PLLA/CNT Composites

In order to explore the influence of modified CNTs on the crystallization properties of PLLA in more detail, we prepared PLLA composites with unmodified CNTs and different concentrations of HBP N103-modified CNTs, which were named PLLA, PLLA/C1, PLLA/C-N0.1, PLLA/C-N0.5, and PLLA/C-N1. The specific concentrations of CNTs in the composites are shown in the following table, Table 1.

Taking sample PLLA/C-N0.1 as an example, the pre-vacuum-dried PLLA 40 g and HBP N103 0.04 g were slowly added to the RM-200C torque rheometer with mixing accessories, and they were plasticized for 5 min at 200 °C in the mixing chamber. The compound was then densified at 50 r/min for 10 min in the chamber at 200 °C. In order to prevent degradation during the dense refining process and reduce the molecular weight, 0.1 wt% phenolic antioxidant (Irganox 1010) was added to the dense refining chamber. In order to facilitate subsequent testing, the samples were made into sheets after refining; the process was carried out as follows: After vacuum drying, the composite was pressed at 200 °C and 10 MPa for 6 min by hot press, during which three rounds of exhaust operation were required. After pressing the composite into sheets with a thickness of 1 mm, the composite was pressed at room temperature with the same pressure for 4 min to ensure the uniformity of thickness of the composite sheet.

### 2.2. Characterization

#### 2.2.1. Fourier-Transform Infrared (FTIR) Spectroscopy

Fourier-transform infrared spectra were recorded on a Nicolet 560 (Nicolet Co., St Peter, MN, USA) Fourier-transform infrared spectrometer at wavelengths ranging from 400 cm^−1^ to 4000 cm^−1^ and a resolution of 4 cm^−1^.

#### 2.2.2. X-ray Photoelectron Spectroscopy (XPS)

An Axis Ultra DLD X Photoelectron spectrometer (Kratos Co., Manchester, Greater Manchester, UK) was used to characterize the C, N, and O contents of the sample. The X-ray source uses a single Al Kα ray with an energy of 1486.6 eV and a voltage of 15 kV. An XPS sensitivity factor quantitative method was used to analyze the contents of carbon and oxygen atoms in the samples calculated by the following formula:

(1) $$C_{x}=\frac{\frac{A_{x}}{S_{x}}}{\frac{\sum A_{i}}{S_{i}}}$$

where *C_x_* is the concentration of the element on the surface, *A_x_* is the proportion of the element peak, *S_x_* is the sensitivity factor of the element peak, and ∑*A_i_*/*S_i_* is sum of ratio of the peak proportion of all elements to the corresponding sensitivity factor. The sensitivity factor of *C*_1*s*_ is 0.278, *O*_1*s*_ is 0.780, and *N*_1*s*_ is 0.477 [24].

#### 2.2.3. Thermogravimetric Analysis (TGA)

The HBP N103 content grafted onto the surface of CNTs was measured using a TG209F1 thermogravimetry (Netzsch Co., Waldkraiburg, Bavaria, Germany) at a rate of 10 °C/min in a nitrogen atmosphere ranging from 25 °C to 800 °C.

#### 2.2.4. Transmission Electron Microscopy (TEM)

The transmission electron microscope Tecnai G2 F20 (FEI, Hillsboro, Ohio, USA) was used to describe the morphology characterization of CNTs and CNTs-N103 with an acceleration voltage of 200 kV and a two-point resolution of 0.24 nm.

#### 2.2.5. Wide-Angle X-ray Diffraction (WAXD)

An Ultima IV X-ray diffractometer (RIGAKU Co., Tokyo, Japan) was used to detect the samples. Cu Kα rays were used as the light source, with wavelength *λ* = 1.54 A, emission voltage of 40 kV, current of 100 mA, and scanning range of 2*θ* = 5–50°.

#### 2.2.6. Differential Scanning Calorimetry (DSC)

DSC 3+ (METTLER TOLEDO Co., Greifensee, Switzerland) was used to conduct differential thermal analysis of the composite materials. The samples of the pre-dried composite materials, amounting to about 4 mg, were chopped up and placed in an aluminum crucible. The samples were heated to 200 °C in nitrogen atmosphere and kept for 8 min to eliminate the thermal history, and then cooled to 25 °C at a rate of 4 °C/min. The calorimetric curve is the crystallization curve and the calorimetric curve is the melting curve. The temperature was raised at rate of 10 °C/min. The crystallinity *X* of PLLA and its composites was calculated according to the following equation [25]:

(2) $$X=\left(\Delta H_{m}-\Delta H_{cc}\right)/\Delta H_{f}^{0}$$

where Δ*H_cc_* is the enthalpy of crystallization during cooling, Δ*H_m_* is the enthalpy of fusion, and Δ$H_{f}^{0}$ is the melting enthalpy of PLLA with infinite crystal thickness [26].

The isothermal crystallization sample preparation was the same as cooling crystallization, but the DSC program setting was different. The sample of about 4 mg was heated to 200 °C and stayed at 200 °C for 8 min. The purpose of this was to eliminate the crystal structure in the sample and create the same thermal history for the composite material. Then, it was cooled to 120 °C at a cooling rate of 50 °C/min, which was maintained for 40 min to record the calorimetric curve.

#### 2.2.7. Polarized Optical Microscopy (PLOM)

The isothermal crystallization process of PLLA and its composites was observed using a Nikon Eclipse LV100N polarizing microscope (Nikon Co., Tokyo, Japan).

## 3. Results and Discussion

### 3.1. Characterization of CNTs

#### 3.1.1. FTIR

**Figure 1:**
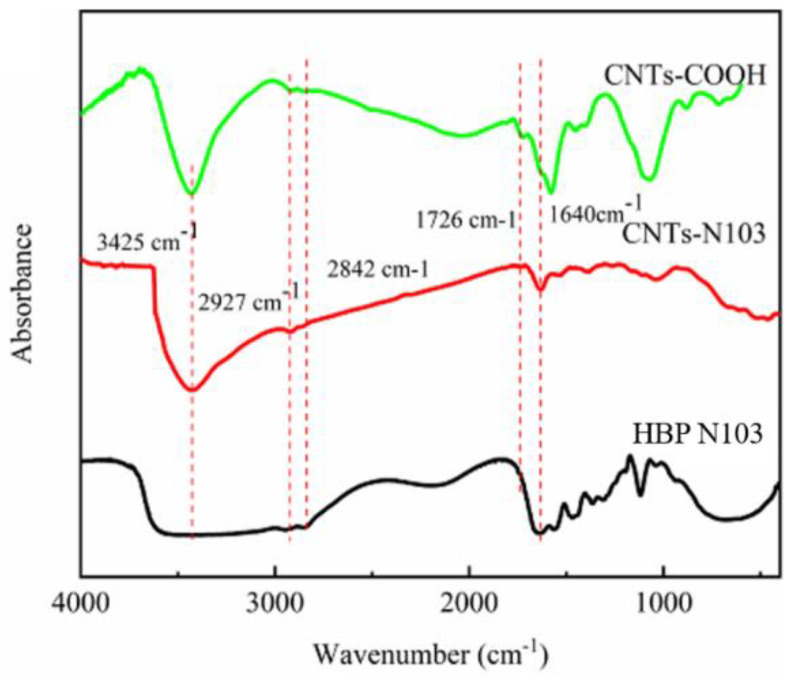
FTIR spectra of CNTs-COOH, CNTs-N103 and HBP N103.

The FTIR spectra of CNTs-COOH, CNTs-N103 and HBP N103 are shown in Figure 1. For HBP N103, there is an obvious first-order amine N-H shear vibration absorption peak of 1640 cm^−1^, and the N-H wide-stretching vibration peak of 3000–3700 cm^−1^ proves that HBP N103 contains a large amount of -NH_2_. In CNTs-COOH, there is a strong and wide absorption peak at 3425 cm^−1^, which is due to the intermolecular hydrogen bond absorption peak caused by -OH. The two peaks at 2927 cm^−1^ and 2842 cm^−1^ are caused by the asymmetric and symmetric vibrations of -CH_3_ and -CH_2_-. Meanwhile, the absorption peak at 1726 cm^−1^ is due to the C=O in the ester group. For CNTs-N103, the weak peak at 1726cm^−1^ is due to the fact that the graft of HBP covered the strength of C=O. The appearance of the absorption peak at 1640 cm^−1^ proves that HBP N103 was successfully grafted onto the surface of the CNTs, and the modification of CNTs is successful. To further confirm the successful modification of CNTs-N103, XPS experiments were carried out.

#### 3.1.2. XPS

**Figure 2:**
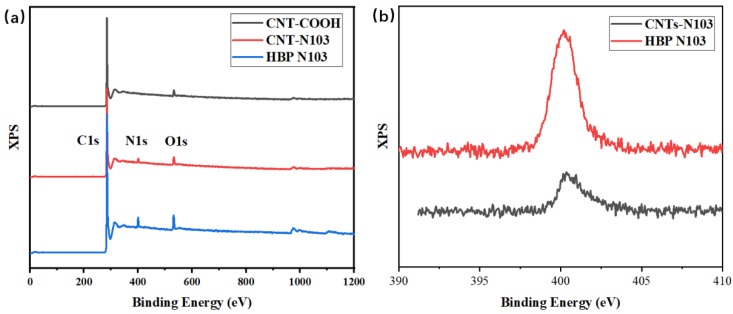
(**a**) XPS survey spectra of CNTs-COOH, CNTs-N103 and HBP N103. (**b**) XPS N*_1s_* survey spectra of CNTs-N103 and HBP N103.

To confirm that the HBP N103 is successfully grafted onto the carbon tube surface, XPS analysis was performed on CNTs-COOH, CNTs-N103 and HBP N103. XPS results are shown in Figure 2. Table 2 shows the relative concentrations of *C*_1*s*_, *N*_1*s*_ and *O*_1*s*_ of the three samples after calculation.

The results revealed that the raw CNTs-COOH does not contain N before modification, as shown in Figure 2a. After the surface modification by HBP N103, which contains sufficient amino, the peak of *N*_1*s*_ appeared in CNT-N103, indicating that HBP N103 was successfully grafted onto the surface of the CNTs. According to the element concentration, it can also be concluded that the successful grafting of HBP N103 makes the CNTs introduce N.

#### 3.1.3. TGA

**Figure 3:**
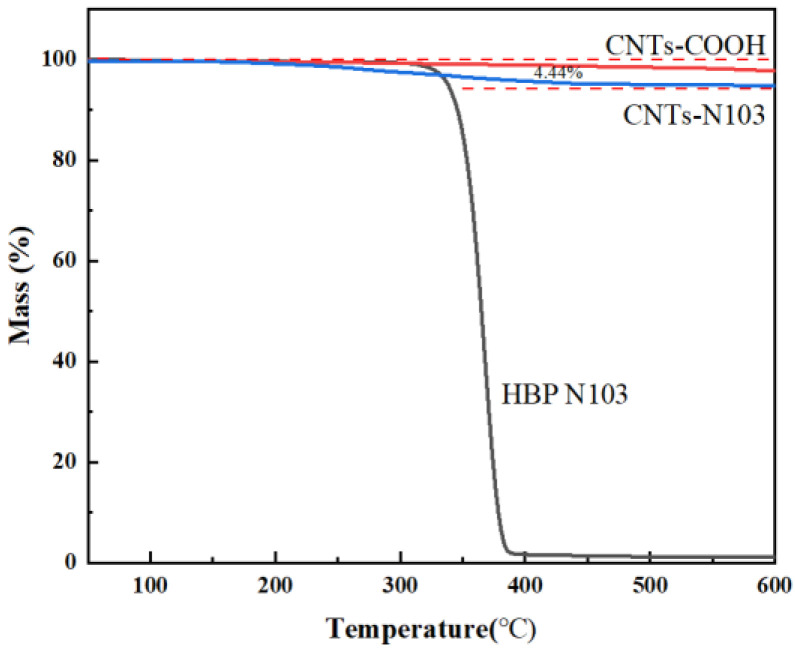
TGA plot of CNTs-COOH, CNTs-N103, and HBP N103.

The TGA results of CNT-COOH, CNT-N103 and HBP N103 are shown in Figure 3. At 320–390 °C, the HBP N103 experiences obvious thermal weight loss, indicating that the HBP N103 is degraded. Meanwhile, compared with the CNTs before modification (CNTs-COOH), the thermal weight loss of the modified CNTs is more obvious. Therefore, it could be determined that HBP N103 was successfully grafted onto the surface of CNTs. Moreover, the thermal loss of the grafted CNTs in the same temperature range is caused by the thermal decomposition of the HBP. Therefore, the TGA analysis shows that the grafting rate of CNTs-103 was 4.44%, the initial temperature of thermal decomposition was 175.4 °C, and the maximum weight loss temperature was 272.9 °C.

#### 3.1.4. TEM

**Figure 4:**
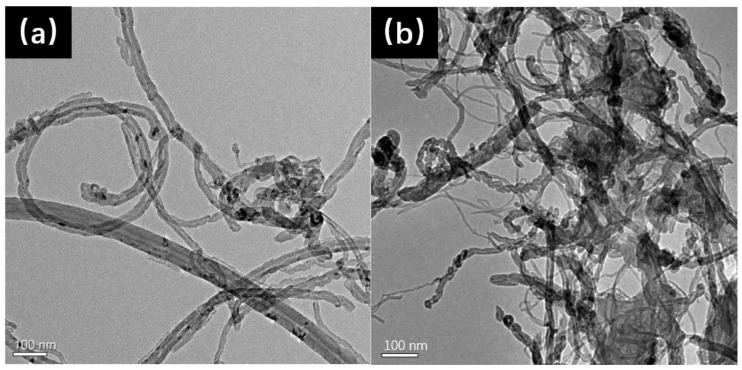
TEM images of (**a**) raw CNTs and (**b**) CNTs-N103.

To directly express the surface morphology of CNTs before and after modification, TEM was performed. The TEM results of unmodified and modified CNTs are shown in Figure 4. Figure 4a shows the surface morphology of a typical unmodified CNT. Figure 4b is the modified CNTs. It can be seen that many black dots appeared on its surface due to graft of HBP N013. In summary, the addition of HBP N103 changes the surface morphology of CNTs.

#### 3.1.5. WAXD

**Figure 5:**
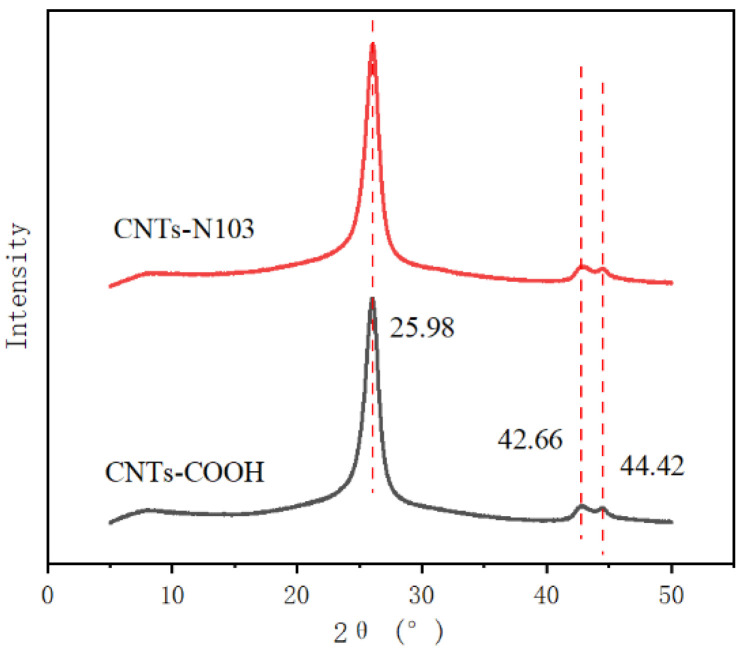
WAXD spectrum of CNTs-N103 and CNTs-COOH.

The diffraction pattern of grafted CNTs under one-dimensional wide-angle X-ray irradiation is shown in Figure 5. No obvious shift of the diffraction peaks can be seen after the modification of the CNTs. The diffraction peaks of (002), (100) and (101) are at 2*θ* angles of 26.00°, 42.74° and 44.42°, respectively, and the crystal plane spacing of (002) was calculated as 3.41 Å. After grafting, the crystal plane spacing did not change significantly at the same position, because the relatively mild grafting reaction conditions would not destroy the structure of the CNTs, so as to ensure the structural integrity of the CNTs, and such a complete structure can be used as a crystal nucleus in PLLA crystallization to shorten the crystallization induction period.

### 3.2. Crystallization Behavior of PLLA/CNT Composites

#### 3.2.1. DSC

**Figure 6:**
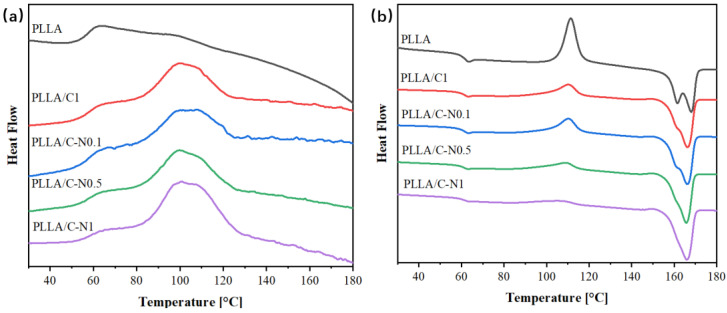
Calorimetric curve of PLLA and PLLA/CNT composites: (**a**) cooling curves and (**b**) subsequent melting curves.

To study the crystallization properties of PLLA and PLLA/CNTs, DSC measurements were conducted. The calorimetric curves of the test results are shown in Figure 6. The calorimetric parameters, including the crystallization peak temperature *T_c_* [27], crystallization enthalpy in the cooling process (shorted in Enthalpy in Table 3) [28], peak temperature of cold crystallization *T_cc_* [29], enthalpy of cold crystallization Δ*H_cc_* in the melting process, the enthalpy of fusion Δ*H_m_* and relative degree of crystallinity *X_c_*, are shown in Table 3 [30].

As shown in Figure 6a, during the cooling process, the pure PLLA sample had no obvious peak, while for samples after the addition of unmodified or modified CNTs, their cooling curves show obvious peaks, indicating that the addition of CNTs promotes the crystallization of PLLA. Meanwhile, crystallization peak temperature *T_c_* and enthalpy of cooling crystallization results listed in Table 3 also suggest that the addition of CNTs greatly promotes the crystallization performance of PLLA. At the same time, by comparing different PLLA/CNT composites, it can be seen that, at the same concentration (PLLA/C-N1 and PLA/C1), the HBP N103-modified CNTs promote the crystallization of PLLA to a higher extent compared with the raw CNTs. On the other hand, in the concentration range of 0.1–1wt%, with the increase in the concentration of CNTs-N103, the PLLA crystallization performance is also improved.

As shown in Figure 6b, during the melting process, all samples show peaks due to glass transition, at about 65 °C, indicating that the addition of unmodified or modified CNTs has no significant influence on the chemical properties of PLLA. Compared with the PLLA/CNT composites, the pure PLLA has a stronger cold crystallization peak, which indicates that the addition of CNTs promotes the crystallization of PLLA.

As shown in Table 3, during the melting process, the peak temperature of cold crystallization *T_c_* and the Δ*H_cc_* decrease, caused by the improvement of crystallization capacity, indicating that the addition of CNTs promotes the crystallization of PLLA, which can be seen in Figure 6b. The Δ*H_m_* of PLLA and PLLA/CNT composites calculated by integrating the melting exothermic peak reveals no obvious regularity, because the samples with lower crystallinity have double peaks caused by recrystallization and crystal transition. Meanwhile, the results of our comparison of the crystallinity of PLLA/CNT composites are similar to those of the cooling crystallization process, where the sample with 1wt% CNTs-N103 has the best promotion effect on the crystallization of PLLA.

**Figure 7:**
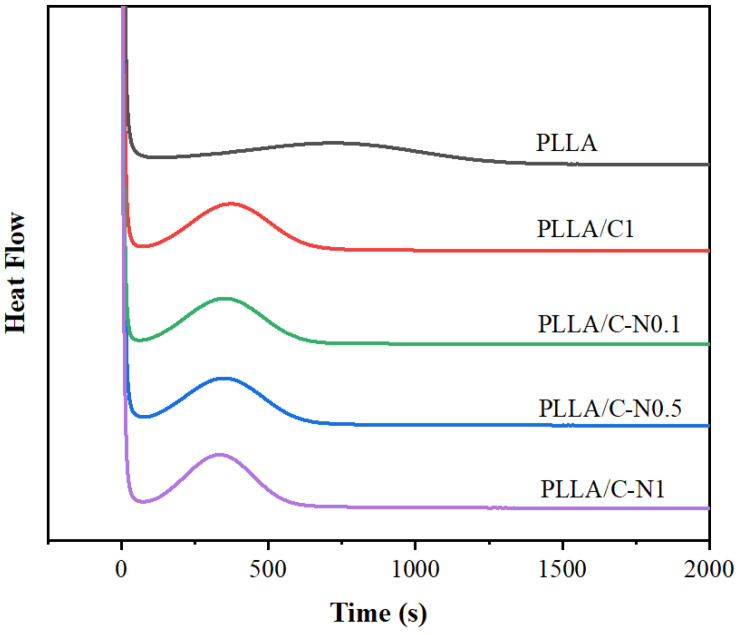
Isothermal crystallization curves of PLLA and PLLA/CNT composites.

**Figure 8:**
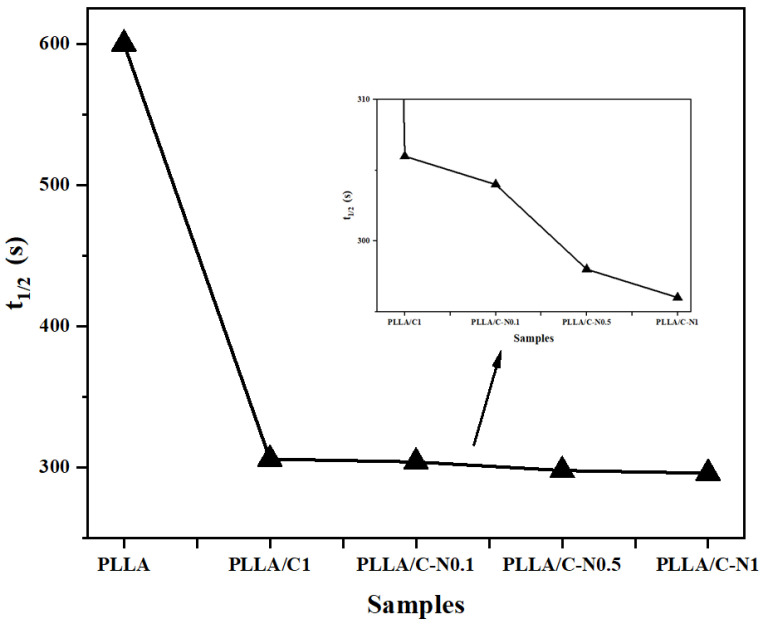
Half crystallization times (t_1/2_) of PLLA and PLLA/CNT composites.

To further explore the influence of CNT modification on the crystallization properties of PLLA, isothermal crystallization experiments were carried out. The DSC results are shown in Figure 7, and the half crystallization times (t_1/2_) are shown in Figure 8. The results show that the addition of CNTs greatly increased the PLLA crystallization ability and shortened the crystal time by nearly half, the most significant being for the PLLA/C-N1, for which the half crystallization time reduced from 600 s to 296 s. Additionally, for different PLLA/CNT composites, the regularity of isothermal crystallization is consistent with that of non-isothermal crystallization. The regulation effect of modified CNTs is better than that of unmodified CNTs at the same concentration. With the increase in the concentration of modified CNTs, its regulation ability also increases.

#### 3.2.2. WAXD

**Figure 9:**
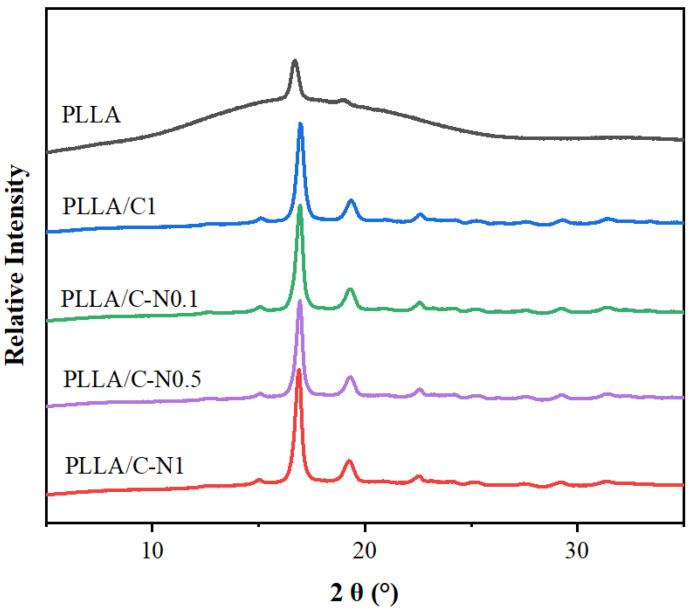
WAXD spectrum of PLLA and PLLA/CNT composites.

The wide-angle X-ray diffraction intensity of PLLA and PLLA/CNT composites during isothermal crystallization at 120 °C for 10 min is shown in Figure 9. It can be seen that the pure PLLA sample has a huge amorphous peak, which indicates that it has a weak crystallization ability. However, when the modified CNTs are added, the amorphous peak disappears and the crystallization peak is sharp, indicating that the crystallization performance of the composites is greatly improved.

#### 3.2.3. PLOM

**Figure 10:**
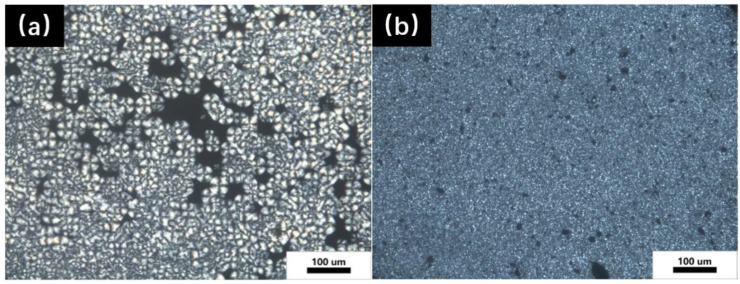

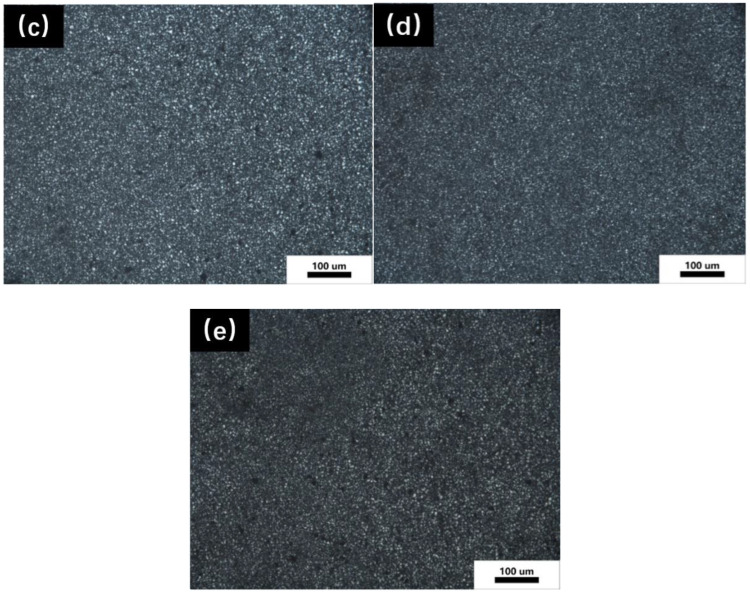
PLOM images of the samples after isothermal crystallization: (**a**) PLLA, (**b**) PLLA/C1, (**c**) PLLA/C-N0.1, (**d**) PLLA/C-N0.5 and (**e**) PLLA/C-N1.

The observations made during the isothermal crystallization process of melting, using a polarizing microscope, are shown in Figure 10. Firstly, the samples were heated to 200 °C for melting, and then were rapidly cooled to 120 °C for isothermal crystallization, and the process of crystallization was observed over time. As shown in Figure 10a, pure PLLA had fewer nucleation points and larger crystals, with a typical grain size of 42.5 μm, which greatly increases the brittleness of PLLA, and its crystallization time is long, about 20 min to complete crystallization. As shown in Figure 10b, after the addition of unmodified CNTs, the nucleation points significantly increased and the grains were refined. The typical grain size is 14 μm, highlighting the presence of many small crystals. Meanwhile, the crystallization time was greatly shortened, and the crystallization was completed in about 8 min. However, some unnucleated points still existed after the crystallization was complete, which is due to the high surface energy of the unmodified CNTs, and the agglomeration phenomenon occurred when it was blended with PLLA. As shown in Figure 10c–e, similar to the addition of unmodified CNTs, the crystallization performance of PLLA composites was significantly improved, the complete crystallization time was greatly shortened, and the grain size as refined, with a typical grain size of 11 μm. Compared with the unmodified CNTs, the modified CNTs showed a better dispersion and the nucleation point location was further increased. The results of PLOM show that the addition of CNTs promotes the crystallization of PLLA, and the modified CNTs have a better promotion than the unmodified CNTs, caused by the better dispersity, which also help to improve the brittleness of PLLA.

## 4. Conclusions

In this paper, CNTs modified with amino HBP were prepared by the amidation reaction. The modification results were characterized by FTIR, XPS, TGA, SEM and WAXD. PLLA and PLLA/CNT composites were prepared and their crystallization and melting kinetics were compared. The results showed that, firstly, the addition of CNTs could greatly improve the crystallization properties of PLLA. Secondly, at the same concentration, the modified CNTs had a better regulation ability on PLLA crystallization than the unmodified CNTs. Moreover, in the concentration range of 0.1–1wt%, with the increase in HBP concentration, the ability of CNTs-N103 to regulate the crystallization of PLLA increased as well.

## Figures and Tables

**Table 1:** The content of CNTs of PLLA/CNT composites.

Content	PLLA	PLLA/C1	PLLA/C-N0.1	PLLA/C-N0.5	PLLA/C-N1
**PLLA (g)**	40	40	40	40	40
**CNTs-N103 (wt%)**	0	0	0.1	0.5	1
**CNTs (wt%)**	0	1	0	0	0

**Table 2:** Element contents of CNTs-COOH, CNTs-N103, and HBP N103.

Sample	*C*_1*s*_ (%)	*N*_1*s*_ (%)	*O*_1*s*_ (%)
CNTs-COOH	96.82	0	3.18
CNTs-N103	93.98	2.63	3.39
HBP N103	65.80	28.52	5.68

**Table 3:** Calorimetric paraments of PLLA and PLLA/CNT composites.

	*Crystallization Process*		*Subsequent Melting Process*			
Sample	*T_c_* (°C)	Enthalpy (J/g)	*T_cc_* (°C)	Δ*H_cc_* (J/g)	Δ*H_m_* (J/g)	*X_c_* (%)
PLLA	99.2	1.8	111.5	28.6	33.8	5.6
PLLA/C1	99.8	17.0	110.4	15.2	35.0	21.2
PLLA/C-N0.1	100.0	16.1	110.3	14.9	34.9	21.3
PLLA/C-N0.5	100.8	19.0	108.8	8.8	32.6	25.4
PLLA/C-N1	101.1	27.3	106.6	7.3	35.2	29.8
